# Spigelian Hernia Following Laparoscopic Hysterectomy: Case Report and Review of the Literature

**DOI:** 10.7759/cureus.99985

**Published:** 2025-12-24

**Authors:** Olga Triantafyllidou, Konstantinos Karkalemis, Alexandros Lazaridis, Fotios Vlahos, Nikolaos Vlahos

**Affiliations:** 1 Second Department of Obstetrics and Gynaecology, Aretaieio University Hospital, Medical School, National and Kapodistrian University of Athens, Athens, GRC; 2 School of Medicine, University of Milano Bicocca, Milan, ITA

**Keywords:** spigelian hernia, surgical case report, total laparoscopic hysterectomy, traumatic small bowel perforation, trocar-site hernia

## Abstract

Spigelian hernia (SH) is a rare type of lateral ventral hernia occurring through the Spigelian fascia, accounting for only 0.1-2% of all ventral hernias. Though commonly considered spontaneous, an increasing number of iatrogenic cases have been reported following laparoscopic surgery, particularly when 10-mm trocar ports are used. Due to the anatomical location between the rectus abdominis and semilunar line, SH can be challenging to detect clinically, often presenting with nonspecific symptoms. This is especially true in gynaecologic laparoscopy, where SH remains an underrecognized complication.

We present the case of a 70-year-old woman who underwent laparoscopic total hysterectomy with bilateral salpingo-oophorectomy for a benign adnexal mass. Two days postoperatively, the patient developed a tender swelling at the trocar site after straining during defecation. CT imaging revealed a loop of small bowel herniated between abdominal wall muscles, with the fascial stitch still visible. During emergency laparoscopy, as we mobilized the small bowel out of the hernia defect, a bowel rupture was identified in the involved segment, leading to fecal peritonitis and necessitating conversion to open surgery. The patient's postoperative course was complicated by severe aspiration pneumonia requiring ICU admission and prolonged mechanical ventilation, as well as a surgical site infection requiring debridement.

This case highlights the diagnostic difficulty and potentially severe complications of trocar-site Spigelian hernias. Even with proper fascial closure, factors such as early postoperative straining, advanced age, and predisposing anatomical defects may contribute to hernia formation. Surgeons should maintain a high level of suspicion in any postoperative patient presenting with localized pain or swelling near trocar sites, particularly lateral to the rectus muscle. Recognizing this uncommon yet potentially serious complication early can ensure early diagnosis and treatment, reducing the risk of morbidity.

This report underscores the need for vigilance in gynaecologic laparoscopic practice and serves as a reminder that even well-closed trocar sites can lead to Spiegel hernia and thus culminate in severe complications.

## Introduction

Spigelian hernia (SH) is an uncommon lateral ventral hernia occurring through the Spigelian fascia, between the rectus abdominis and semilunar line [1]. The actual frequency of SH in the general population is uncertain, since many individuals never develop symptoms. It represents 0.1-2% of ventral hernias and is more frequent in women >60 years [2,3]. Although traditionally considered spontaneous, iatrogenic Spigelian hernias have been increasingly reported after laparoscopic surgery, particularly at ≥10-mm trocar sites [4,5].

The Spigelian hernia sac tends to spread laterally between internal and external oblique muscles, and it is characterized by an intact external oblique aponeurosis covering the hernia [6], making the clinical signs often subtle. Postoperative hernia represents a potential complication of laparoscopic procedures, most frequently arising at trocar insertion sites. Misdiagnosis as hematoma or seroma is common, delaying definitive treatment and increasing the risk of bowel incarceration, strangulation, and perforation. SH after laparoscopy in general surgery is well described [7]; however, there are still few publications of SH after gynaecological laparoscopic procedures [8,9]. We present a rare severe case of trocar-site Spigelian hernia following laparoscopic hysterectomy, complicated by bowel necrosis, aspiration pneumonia, ICU stay, and wound infection, and provide a review of the literature.

## Case presentation

A 70-year-old woman with a BMI of 23 and a history of diaphragmatic hernia and congenital left torticollis underwent laparoscopic total hysterectomy with bilateral salpingo-oophorectomy for a large right adnexal benign cyst 10cm in diameter. Pneumoperitoneum was performed using an open technique and a Hasson trocar. Three additional trocars were placed (Unimicro, bladed, auto-shield, double-seal, key switch auto-locking system (Unimicro Medical Systems (Shenzhen) Co., Ltd., Shenzhen, China)): a 5 mm trocar at the right lower quadrant (RLQ) and a 10 mm trocar at the left lower quadrant (LLQ), approximately 2 cm medial and 2 cm superior to the anterior superior iliac spine (ASIS), laterally to the rectus abdominis muscle, and a 5 mm trocar in the midline suprapubicly. A 10 mm trocar was placed in the LLQ for the removal of the ovaries inside a 10 mm endoscopic retrieval bag. After completing the hysterectomy, the fascial defect was closed with a #1 Vicryl suture under direct visualization. She was discharged the following day.

Two days postoperatively, after straining during defecation, the patient noticed a 2-3 cm tender lump close to the left trocar site. The patient didn’t report nausea or pain but was referred back to the hospital for further investigation. Laboratory tests revealed no evidence of inflammation, with both C-reactive protein and white blood cell count within normal ranges (WBC 9x10³/µL and CRP 2.2 mg/dl (normal range <5 mg/dl)). She had passed stool that morning. An abdominal ultrasound was performed by the resident radiologist on call, which suggested a small hematoma at the LLQ. Accordingly, there was no clinical suspicion of bowel obstruction at that time, and the patient was hospitalized for conservative management with intravenous fluids and a plan for new lab tests the following day. She remained stable for 24 hours.

**Figure 1:**
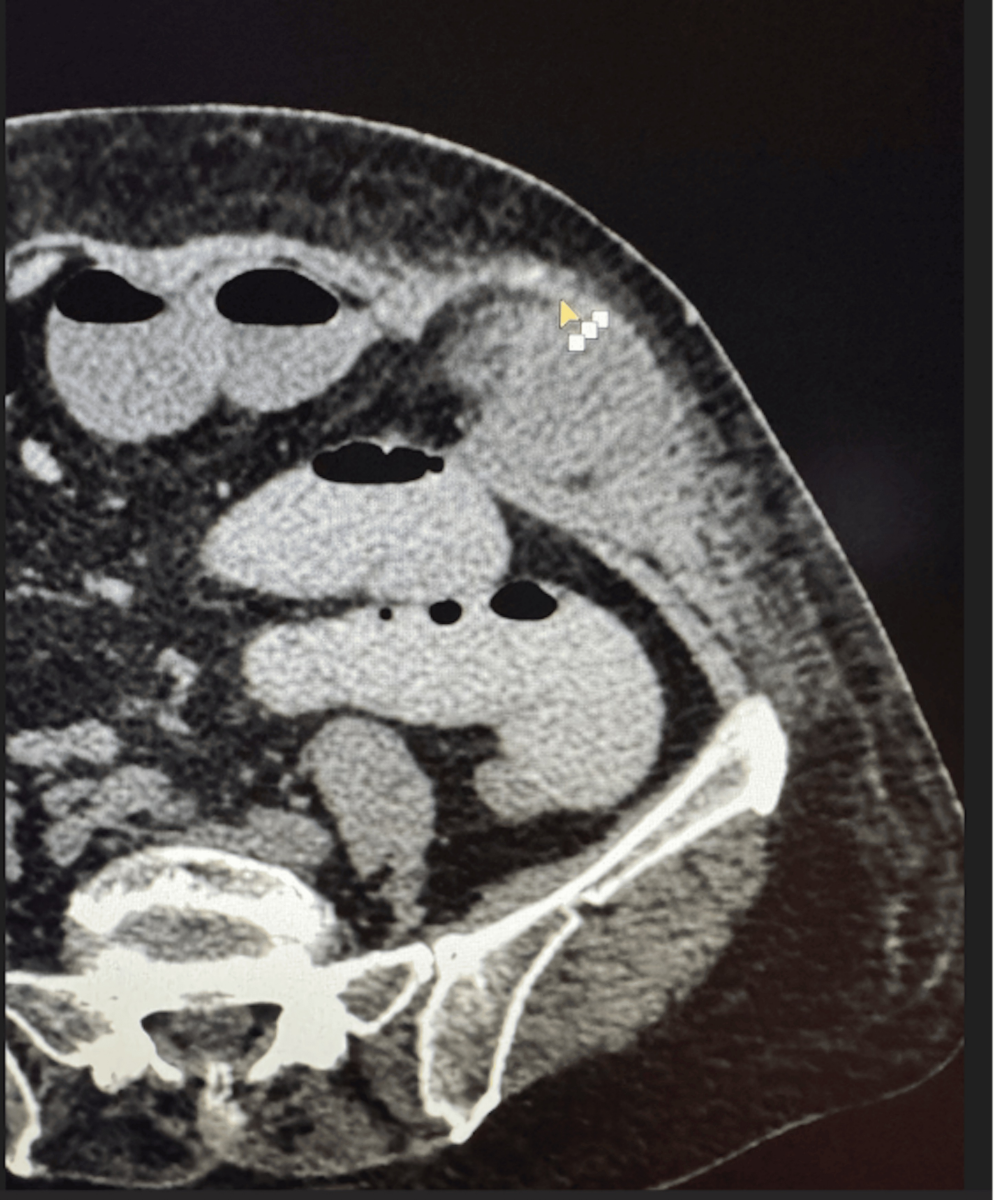
Abdominal CT with oral contrast: Spigelian hernia at the left trocar site The arrowhead highlights the placed suture and the intact anterior rectus sheath.

Subsequently, 24 hours post-admission, she developed severe left lower quadrant pain and nausea without vomiting. On examination, bowel sounds were present, and there was no distension. Follow-up blood tests demonstrated that inflammatory markers remained stable (WBC 9.6x10³/µL and CRP 3.1 mg/dl (normal range <5mg/dl)), with no significant change compared to the previous results. CT with oral contrast revealed a loop of small bowel trapped between abdominal wall muscles but beneath the intact fascia. It is important to note that the loop of bowel was not located at the trocar port-site incision, and the fascial stitch was visible (Figure 1). The hernia orifice was visible at the level of the transverse abdominal muscle aponeurosis.

She was scheduled for immediate laparoscopic repair and reduction. Prior to surgery, the patient received antibiotic prophylaxis with ciprofloxacin and metronidazole, and a nasogastric tube was inserted. We elected to perform a laparoscopy rather than a laparotomy to allow rapid re-entry through the existing laparoscopic incisions to initially assess the bowel. Intraoperatively, a segment of small bowel was found to be herniated through the peritoneal defect in the left lower quadrant. During manipulation at the time of laparoscopy, the loop ruptured, resulting in faecal peritonitis. Conversion to laparotomy was performed by extending the incision in the area of the left lower quadrant, which clearly revealed an intact fascial closure at the trocar site. A 15 cm segment of necrotic bowel was resected with primary site-to-site anastomosis. A 27 Fr silicone closed-suction drain was placed in the pouch of Douglas through the suprapubic trocar incision site. The abdominal wall overlying the site of the hernia was approximated with a running full-thickness #1 PDS (polydioxanone) suture, and the skin was approximated with staples.

Her postoperative course was complicated by severe aspiration pneumonia two days after the re-operation, presenting with high fever, necessitating admission to the intensive care unit (ICU). She was septic and required endotracheal intubation and mechanical ventilation for 20 days, during which time her fever gradually resolved, and ventilatory settings were progressively reduced in response to the improved respiratory function. During her ICU stay, she was treated with triple antimicrobial therapy consisting of piperacillin-tazobactam, metronidazole, and antifungal therapy with fluconazole. She also developed a surgical site infection in the area of the laparotomy incision, which required wound debridement and wet-to-dry dressings, allowed to close by secondary intention. Despite her complicated postoperative course and prolonged recovery, she was eventually discharged in good condition on the 40th postoperative day. At the six-month follow-up, she remained asymptomatic with no recurrence.

## Discussion

This is the fifth reported case of SH presenting after gynecologic laparoscopic surgery [4,9-11]. Trocar site hernias complicate 0.23-1% of laparoscopies [4]. Specifically, in laparoscopic gynaecologic procedures, the incidence of incisional hernias at assistant port sites varies from 0.23% to 3.1% based on the use of a 10 mm or a 12 mm trocar, respectively [12,13]. The herniation of Spigelian fascia in adults during laparoscopy is very rare (2% of all hernias) [7]. In gynaecology, in particular, the incidence of SH is even lower. This is a rare case of SH after gynecologic laparoscopic surgery. Accurate differentiation between an SH and a trocar site hernia is critical, as they represent distinct pathological entities with different etiologies. A review of the four previously reported cases of spigelian hernia after gynecologic laparoscopy is presented in Table 1. 

**Table 1:** Review of reported cases of SH after gynecologic laparoscopic surgery SH: Spigelian hernia; RLQ: right lower quadrant; LLQ: left lower quadrant

Author	Year	Age	Operation	Onset of postoperative symptoms	Symptoms	Site of SH	Management	Mesh placement
Yamamoto et al. [10]	2011	43	Laparoscopic Hysterectomy	Day 4	Nausea, vomiting, abdominal tenderness	RLQ	Laparotomy - Viable small bowel reduced in abdomen – no resection / no mesh	No mesh
Bassi et al. [11]	2012	60	Laparoscopic Bilateral Salpingo-Oophorectomy	Day 2	Abdominal pain, nausea, and abdominal mass	RLQ	Laparotomy -Viable small bowel reduced in abdomen – no resection / no mesh	No mesh
Kamel et al. [4]	2012	68	Laparoscopic Right Oophorectomy	Day 14	Abdominal pain and repeated vomiting, bile stained, abdominal distension.	LLQ	Viable small bowel reduced in abdomen – no resection	Prolene mesh placement
Ussia et al. [9]	2017	35	Laparoscopic Excision of Superficial/Deep/Ovarian Endometriosis	Day 7	Pain in the right fossa and physical activity, dyspareunia diagnosed as nerve entrapment at the time	RLQ	3rd diagnostic laparoscopy 4 years later – no resection	No mesh

A SH is a primary intraparietal hernia that protrudes through the Spigelian aponeurosis, and, crucially, the external oblique aponeurosis remains intact with the intact fascial closure at the trocar site, often making it difficult to detect. In contrast, a laparoscopic trocar site hernia is an incisional hernia where all layers of the abdominal wall, including fascia, are breached at a previous surgical port site, while their presentation may be similar. The development of SH may be partly attributable to pneumoperitoneum in laparoscopic procedures when a latent defect in the Spigelian fascia is present [9,14]. Other mechanisms include fascial disruption, muscle splitting, increased intra-abdominal pressure, chronic obstructive pulmonary disease (COPD), obesity with rapid weight loss, multiple pregnancies, and patient-related factors such as advanced age (>60 years old), female sex, previous pregnancies, and high intra-abdominal pressure during delivery, and reduced abdominal wall strength [3,15,16]. Despite fascial closure, herniation may occur, particularly after early postoperative straining, as in this case. It is imperative to clarify that an SH is a distinct primary abdominal wall pathology that appears after laparoscopy. Ιt is not an iatrogenic complication of trocar placement [4,5,14]. A systematic review of over 200 SH cases suggests a slight predominance on the left side, as in our case [17].

Diagnostic challenges

Usually, the defects of SH are asymptomatic. Clinical presentation is often nonspecific, making diagnosis challenging; nevertheless, Kamel et al. (2012) [4] highlighted the importance of surgical repair upon detection regardless of symptoms due to the relatively high risk of strangulation. Tonouchi et al. categorized the symptomatology of these patients in three categories, the early-onset, which usually occurred immediately or within 12 days of the operation and includes a small-bowel obstruction, such as in our case, the late-onset type occurring several months later, mostly with local abdominal bulging but no bowel obstruction and the special type that is defined by the protrusion of the intestine and/or omentum [18]. On the first, early-onset type, patients usually present with an acute abdomen due to small-bowel obstruction or a mass protruding from the abdominal wall around the trocar site. Sonography may fail to detect intraparietal hernias, leading to misdiagnosis as hematomas or seromas. Kamel et al. emphasized diagnostic pitfalls and under-recognition of this hernia type in gynaecologic practice [4]. MRI and CT are superior, visualizing herniated bowel beneath intact fascia and confirming fascial closure. In this patient, CT with oral contrast established the diagnosis after ultrasound failed.

Surgical considerations and complications

Spigelian hernias carry a high risk of incarceration (up to 17%) and strangulation [3]. This is primarily attributed to the relatively small and rigid defect in the Spigelian fascia compared with the size of the hernia contents. Surgical repair is mandatory as early as possible for the prevention of bowel necrosis [5]. Traditionally, hernia repair involves open anterior herniorrhaphy with direct muscle approximation, often supported by mesh or other prosthetic materials. Laparoscopic techniques are also used, preferably through a totally extraperitoneal approach, or via an intraperitoneal route, when additional surgical procedures are performed during the same operation [16,17,19]. Laparoscopic repair allows good exposure, but reduction of necrotic or tightly incarcerated bowel is hazardous, as rupture may occur. Still, a risk of recurrence exists in approximately 3% of reoperations [19]. Surgeons must maintain a low threshold for conversion and be prepared for bowel resection. On the other hand, the placement of laparoscopic trocars itself, especially the use of a 10 mm trocar, can be the cause of the iatrogenic SH. For this purpose, the use of an atraumatic round trocar instead of a triangular traumatic tip is recommended to decrease the fascial and muscular dissection [9,20].

## Conclusions

Spigelian hernias are more common than generally suspected and may remain undiagnosed, especially in the presence of atypical symptoms. However, trocar-site Spigelian hernia is a rare but severe complication of laparoscopic surgery, even when fascial closure is performed. We should maintain a high index of suspicion for SH in gynaecological practice in women presenting with localized pain lateral to the rectus muscle, following laparoscopy. Elderly women are at particular risk. Any postoperative port-site swelling warrants high suspicion, and cross-sectional imaging should be obtained. Early surgical intervention is essential. Surgeons must be prepared for open conversion and bowel resection when bowel viability is compromised. Delay can lead to catastrophic complications. Our case illustrates how delayed diagnosis can culminate in severe complications - bowel necrosis, perforation, faecal peritonitis, aspiration pneumonia, prolonged ICU stay, and wound infection. This highlights the need for early suspicion, rapid imaging with CT scans and intravenous contrast, if available, and definitive management.
